# Large language model triaging of simulated nephrology patient inbox messages

**DOI:** 10.3389/frai.2024.1452469

**Published:** 2024-09-09

**Authors:** Justin H. Pham, Charat Thongprayoon, Jing Miao, Supawadee Suppadungsuk, Priscilla Koirala, Iasmina M. Craici, Wisit Cheungpasitporn

**Affiliations:** ^1^Mayo Clinic College of Medicine and Science, Mayo Clinic, Rochester, MN, United States; ^2^Department of Nephrology and Hypertension, Mayo Clinic, Rochester, MN, United States; ^3^Faculty of Medicine Ramathibodi Hospital, Chakri Naruebodindra Medical Institute, Mahidol University, Samut Prakan, Thailand; ^4^Department of Internal Medicine, Mayo Clinic, Rochester, MN, United States

**Keywords:** large language model, ChatGPT, inbox messages, triage, patient care, patient communication, artificial intelligence

## Abstract

**Background:**

Efficient triage of patient communications is crucial for timely medical attention and improved care. This study evaluates ChatGPT’s accuracy in categorizing nephrology patient inbox messages, assessing its potential in outpatient settings.

**Methods:**

One hundred and fifty simulated patient inbox messages were created based on cases typically encountered in everyday practice at a nephrology outpatient clinic. These messages were triaged as non-urgent, urgent, and emergent by two nephrologists. The messages were then submitted to ChatGPT-4 for independent triage into the same categories. The inquiry process was performed twice with a two-week period in between. ChatGPT responses were graded as correct (agreement with physicians), overestimation (higher priority), or underestimation (lower priority).

**Results:**

In the first trial, ChatGPT correctly triaged 140 (93%) messages, overestimated the priority of 4 messages (3%), and underestimated the priority of 6 messages (4%). In the second trial, it correctly triaged 140 (93%) messages, overestimated the priority of 9 (6%), and underestimated the priority of 1 (1%). The accuracy did not depend on the urgency level of the message (*p* = 0.19). The internal agreement of ChatGPT responses was 92% with an intra-rater Kappa score of 0.88.

**Conclusion:**

ChatGPT-4 demonstrated high accuracy in triaging nephrology patient messages, highlighting the potential for AI-driven triage systems to enhance operational efficiency and improve patient care in outpatient clinics.

## Introduction

The advancement and dissemination of artificial intelligence (AI) in recent years has generated considerable interest about its implications for healthcare (Jiang et al., 2017; Alowais et al., 2023; Bajwa et al., 2021). Previous studies have demonstrated the potential of AI in various clinical applications, from diagnostic support to personalized treatment recommendations (Chen et al., 2021; Liu et al., 2023). AI-based systems have been used in radiology to detect anomalies in medical imaging, in pathology to classify diseases, and in general practice to aid clinical decision-making (Kelly et al., 2022; Syed and Zoga, 2018; Bera et al., 2019; Cohen et al., 2021; Giordano et al., 2021). Beyond these clinical applications, there has also been growing recognition of their potential application toward streamlining administrative tasks, such as patient scheduling and follow-up management.

One promising implementation in this regard is the management of electronic health record (EHR) inbox messages. Addressing large volumes of patient messages has become a common challenge for healthcare providers across all specialties in recent years (Murphy et al., 2019; Escribe et al., 2022). The added workload of managing a busy electronic health record (EHR) inbox adds significant burden to the regular demands of clinical practice and is frequently cited as a major contributor to provider burnout (Murphy et al., 2019; Margolius et al., 2020; Budd, 2023). This has been especially true in the field of nephrology, where providers must regularly attend to messages spanning a wide range of medical complexity (Nair et al., 2022). Some queries require immediate attention, such as those concerning significant kidney injury or hypertensive emergencies, while others are related to indirect patient care needs. EHR inbox triaging can be used to organize messages based on clinical acuity, allowing clinicians to attend to them more efficiently and ensuring the most urgent needs are prioritized.

Large language models (LLMs) such as ChatGPT may be uniquely suited to fill this role due to their proficiency in interpreting natural language inputs. LLMs have already demonstrated considerable utility in several healthcare applications (De Angelis et al., 2023; Li et al., 2024; Dave et al., 2023; Cascella et al., 2023). Recent studies have shown that they can also be used to triage clinical cases based on encounter documentation in emergent settings (Williams et al., 2024; Frosolini et al., 2024). To our knowledge, the ability of LLMs to accurately triage patient communications has not yet been explored. In the present study, we evaluate the accuracy and reliability of ChatGPT in categorizing simulated inbox messages from patients of an outpatient nephrology clinic by urgency. In doing so, we aim to provide insight into the potential utility and reliability of LLMs in enhancing operational efficiency by automating the triage process in real-world clinical settings.

## Materials and methods

### Dataset

Two nephrologists in our team (CT and WC) wrote a total of 150 simulated patient inbox messages based on cases encountered in an outpatient nephrology clinic. The messages were designed to represent a balanced distribution of urgency levels, with 50 messages simulating medical emergencies necessitating immediate attention, 50 simulating urgent matters requiring medical attention within 48 to 72 hours, and 50 simulating non-urgent matters that could be addressed electively during a routine outpatient appointment. The urgency level of each message was verified and agreed upon by both nephrologists. The messages were written to include a wide variety of clinical scenarios encompassing the spectrum of medical urgency. Simulated crises included subjective reports of severe symptoms such as excruciating pain, difficulty breathing, and severe headaches. Less-urgent messages involved patients seeking advice on lifestyle changes, reporting new mild symptoms, sharing concerns about the effectiveness of their current medications, inquiring about alternative therapies and new treatment options, and wanting to discuss the long-term health impacts of their disease. Patients often expressed their concerns with varying levels of urgency, fear, and confusion, seeking reassurance and guidance from their healthcare providers. Scenarios were patient-centered, with many messages reflecting anxiety about symptoms, treatment efficacy, and potential complications.

### ChatGPT triage

The messages were submitted to ChatGPT-4, the latest version of ChatGPT that had been released by OpenAI at the time of the study (May 2024). ChatGPT was first given the following prompt: “I am going to provide you with messages from patients to their physician. Please categorize each message into one of three categories: emergency (requires immediate medical attention), urgent (must be addressed in 48 to 72 hours), or non-urgent (can be addressed during a regularly scheduled appointment).” All 150 inbox messages were entered into the ChatGPT interface in a randomized fashion one by one, and the categorization responses were recorded for each. To assess the internal consistency of ChatGPT’s responses, this process was repeated 2 weeks later, with the sequence of the messages having been re-randomized prior to submission.

### Quantitative analysis

ChatGPT responses were graded as correct (agreement with intended triage level), overestimation (higher level of priority than required), or underestimation (lower level of priority than required). ChatGPT’s responses between the first and second trials were compared using McNemar’s test, and Cohen’s kappa statistic (*κ*) was calculated to determine intra-rater agreement. Kappa values, which range from 0 to 1 for agreement, were interpreted using the following thresholds: 0.01–0.20 (slight agreement), 0.21–0.40 (fair agreement), 0.41–0.60 (moderate agreement), 0.61–0.80 (substantial agreement), and 0.81–1.00 (near-perfect agreement). Data were managed and analyzed using R statistical software (version 4.1.0).

### Qualitative analysis

A qualitative analysis was conducted alongside the quantitative analysis to identify any apparent patterns in ChatGPT’s responses, as well as sources of erroneous triage. Each response was independently reviewed by all members of our team, with particular attention given to cases of triage misclassification.

## Results

**Figure 1:**
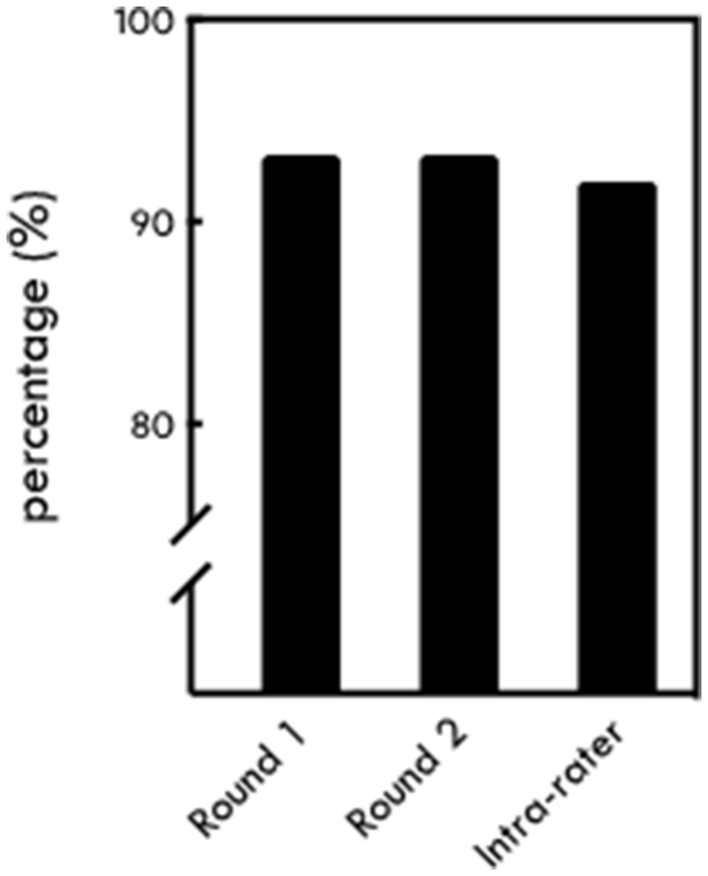
Overall percentage of messages triaged correctly by ChatGPT in round 1 and round 2, as well as the percentage of agreement between rounds (intra-rater).

**Figure 2:**
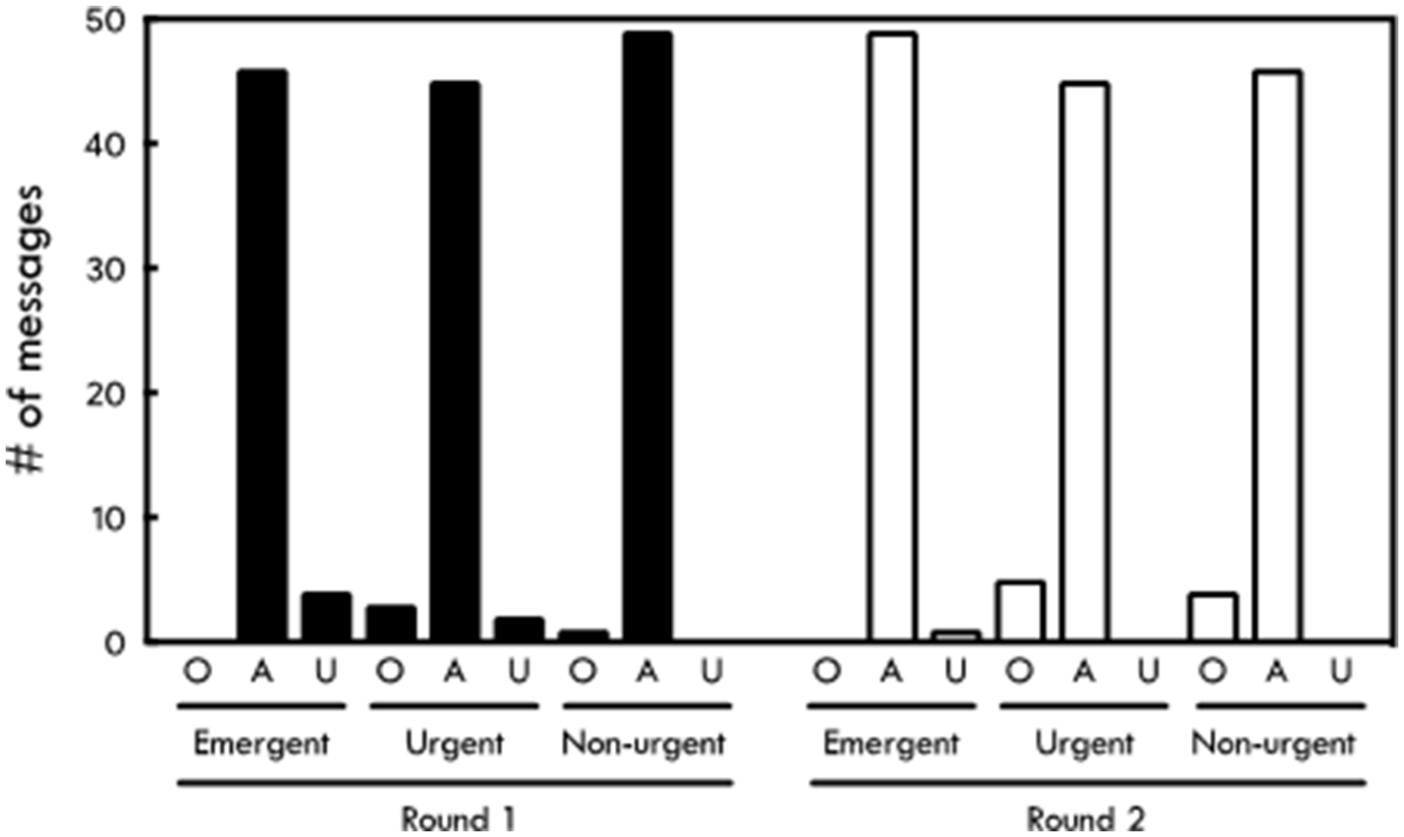
Accuracy of ChatGPT triage results compared to physician triage by message category. O, overestimation; A, agreement; U, underestimation.

ChatGPT correctly triaged 140 out of 150 messages (93%) in the first trial (Figure 1). It correctly triaged 49 non-urgent messages (98%), 45 urgent messages (90%), and 46 emergent messages (92%) (*p* = 0.19) (Table 1). It overestimated the priority of 1 non-urgent message and 3 urgent messages while underestimating the priority of 2 urgent messages and 4 emergent messages (Figure 2).

**Table 1:** Accuracy of ChatGPT for triage of patient inbox message.

	1st trial	2nd trial
All	140 (93%)	140 (93%)
Non-urgent	49 (98%)	46 (92%)
Urgent	45 (90%)	45 (90%)
Emergent	46 (92%)	49 (98%)

In the second trial, ChatGPT also correctly triaged 140 (93%) messages (Figure 1). Forty-six of these were non-urgent (92%), 45 were urgent (90%), and 49 were emergent (98%) (*p* = 0.19). It overestimated the priority of 4 nonurgent messages and 5 urgent messages, while underestimating the priority of 1 emergent message (Figure 2). There was no difference in the accuracy of responses between the first and second trials (*p* = 1.00). The intra-rater agreement was 92% with a Kappa agreement of 0.88.

## Discussion

The advent of EHR-based patient portals and clinical messaging systems has given patients the freedom to communicate with members of their healthcare team at will, allowing them an unprecedented level of access to their providers. This marks a tremendous step forward in strengthening the patient-provider relationship but has had the unfortunate consequence of significantly contributing to clinician workload and burnout. EHR inbox management could be made more efficient and less burdensome through message triage, which would improve patient outcomes by ensuring urgent needs are attended to immediately and medical resources are directed appropriately. Triage is a critical component of clinical practice, but traditional triage methods rely heavily on provider clinical judgment which can be resource-intensive and subject to variability. Currently, there are no widespread systems in place for the automated triaging of EHR communications, meaning providers typically respond to messages as they are able to and in the order in which they are received. Some groups have found success in tackling this issue by having nursing and support staff manage and triage patient communications (Halstater and Kuntz, 2023). To our knowledge, this is the first study to demonstrate the potential for LLMs to fill this role.

The primary outcome measures were the accuracy of ChatGPT’s triage decisions and the intra-rater consistency observed between trials. ChatGPT demonstrated near-perfect overall agreement with physicians in triaging simulated nephrology patient inbox messages (*κ* = 0.9), as well as near-perfect internal consistency across time (*κ* = 0.88). Triage overestimation was more common than underestimation across the two trials overall, though there were more cases of underestimation observed in the first trial. Our qualitative analysis did not reveal any obvious trends in terms of subject matter or recurrent errors related to specific scenarios. ChatGPT demonstrated solid clinical judgement overall, without any evidence of hallucination, fabrication, or blatant deficiencies in its medical knowledge or decision making. It provided sound rationale for each of its responses, and each case of miscategorization was due to slight discrepancies between its interpretation of the urgency of a message and that of the nephrologists on our team. All cases of miscategorization involved adjacent triage levels (e.g., emergent instead of urgent, or urgent instead of non-urgent). There were no incidences of “skipping” triage levels (i.e., categorizing an emergent message as non-urgent, or vice versa). Although categorizations were fairly accurate across all three triage levels in both trials, ChatGPT had the lowest accuracy for urgent messages overall. This could be explained by the fact that, as the “middle” category, urgent messages could be miscategorized in either direction while emergent messages could only be underestimated and nonurgent ones could only be overestimated. Overall, these findings suggest that advanced LLMs such as ChatGPT have potential to assist with triaging patient communications accurately and reliably in clinical settings, allowing clinicians to address them more efficiently. This can significantly reduce the administrative burden on clinical staff, allowing them to devote more time to direct patient care.

The integration of AI in clinical practice represents a transformative phase in healthcare. It has been able to achieve diagnostic accuracy comparable to human clinicians in various medical specialties, such as dermatology and ophthalmology (Behrmann et al., 2023; Du-Harpur et al., 2020; Ting et al., 2019). The potential role for AI in patient-facing roles such as automated triage has generated interest amongst healthcare professionals, but is not without caveats (Townsend et al., 2023). A key concern is data privacy. AI systems require access to large volumes of patient data to function effectively, which raises significant concerns about the security and confidentiality of sensitive health information. Ensuring that patient data is protected from unauthorized access, breaches, and misuse is paramount. Healthcare providers and developers of AI systems must adhere to stringent data protection regulations and implement robust encryption and data anonymization techniques to safeguard patient information (De Angelis et al., 2023). Algorithmic bias is another critical issue. LLMs are trained on data that may contain inherent biases, which can lead to unfair or inequitable treatment of certain patient populations. If the training data is not representative of certain demographics, the model could make less accurate or biased decisions for underrepresented groups. It is essential to ensure that these systems are trained on diverse datasets and that continuous monitoring and auditing are in place to identify and correct any biases that may emerge over time. Guidelines and regulations must be established to govern the use of AI prior to its widespread adoption in clinical practice in order to safeguard patient interests and promote ethical and equitable standards (Parikh et al., 2019; Amann et al., 2020; Čartolovni et al., 2022). Importantly, healthcare providers and patients alike must understand how decisions are made by AI systems to maintain trust and accountability (De Angelis et al., 2023; Williamson and Prybutok, 2024). One way in which this can be achieved is through the use of explainable AI (XAI) techniques that provide clear, understandable explanations for AI-generated recommendations.

While AI can be a powerful tool for supporting clinical decision-making, it should not replace the expertise and judgment of healthcare professionals. It is important to emphasize the role of AI as a complementary tool rather than a replacement for human decision-making. Clinicians should be trained to critically evaluate AI outputs and integrate them with their clinical expertise to ensure the best outcomes for patients. As AI systems become more adept at handling routine tasks like triaging patient communications, healthcare professionals may find that their roles shift from performing these tasks to overseeing and managing AI-driven processes. This shift could lead to a redefinition of professional responsibilities, where clinicians are required to focus more on complex decision-making, patient interactions, and the interpretation of AI-generated insights rather than routine administrative duties. Interdisciplinary teams in which data scientists, technologists, and clinicians work closely together to ensure that AI-based tools are implemented effectively will likely become more commonplace. This collaboration could lead to the development of new roles within healthcare organizations, focused on the integration and management of AI systems. This transformation also presents challenges, such as the potential risk that healthcare professionals may become dependent on these tools. It is essential to ensure that clinicians remain actively involved in the decision-making process, continue to exercise their judgement, and engage in continuous training and upskilling to keep up with the latest developments in technology. Future healthcare professionals will need to be equipped with the skills to work alongside this technology, including an understanding of data science, AI ethics, and the principles of machine learning. Integrating these topics into medical curricula will help prepare the next generation of clinicians for a healthcare environment increasingly shaped by AI.

This study adds to the growing recognition of the potential for AI to streamline numerous aspects of healthcare administration. LLMs similar to ChatGPT could potentially be used to automate appointment scheduling based on urgency and specialty needs, optimize clinic workflows, and provide personalized follow-up recommendations by analyzing patient records and treatment outcomes. Such systems would not only enhance operational efficiency but also aim to improve patient satisfaction by ensuring timely and appropriate care delivery. Exploring applications such as portal message triage provides a more comprehensive understanding of AI’s potential to revolutionize various facets of healthcare management. Rigorous testing and validation protocols must first be established to ensure the reliability and safety of AI tools before they are deployed in clinical settings. These protocols should include diverse real-world scenarios to better simulate the complex and varied nature of patient communications. Ongoing monitoring and evaluation are essential to promptly identify and correct any issues that may arise in operational use, such as biases or errors in triage decisions.

### Limitations

Our study involved a relatively limited sample (*n* = 150) of simulated messages written by professional nephrologists instead of real-world data. The messages were written to clearly depict specific clinical scenarios in nephrology for the sole purpose of rapid triage, and may not reflect the true breadth and complexity of real-world communications which are often more ambiguous, nuanced, and difficult to interpret. Though promising, our results are limited by the simulated nature of our study design and the findings should be interpreted with caution when considering their potential applicability in real-world settings.

### Future directions

To further validate our findings, future studies should aim to incorporate real-world patient communications from across a diverse range of clinical scenarios. This could allow researchers to capture the full spectrum of complexities and variabilities inherent in actual clinical practice such as ambiguous patient presentations, varying communication styles, and the presence of extraneous or conflicting information that can influence decision-making processes. Studies that measure the outcomes of incorporating LLMs into EMR-based communications are also needed to assess their benefit and justify their adoption. Key metrics to consider include time saved for clinicians, improvements in clinical efficiency, reduction in patient wait times for responses, and improvement in patient satisfaction.

The generalizability and utility of our approach in everyday clinical practice can be further assessed by studying broader and more complex datasets that include other medical subspecialties. Examining the applicability of our approach in these different contexts could reveal important insights into how to further optimize triage and communication in specific settings. By conducting studies that compare the effectiveness of this approach in different specialties, best practices could be identified that could be tailored to meet the specific demands of each field, ultimately improving patient outcomes and the efficiency of healthcare delivery.

## Conclusion

This study demonstrates the potential of ChatGPT-4 in accurately triaging patient inbox messages, suggesting that LLMs can be a valuable tool in clinical practice to enhance operational efficiency and patient care. Further research is warranted to explore its application in other areas of healthcare and to address ethical considerations. Future studies should involve larger datasets and more diverse clinical settings to validate these findings and assess the broader applicability of ChatGPT-4. Additionally, developing frameworks to ensure the ethical use of AI in healthcare will be crucial in leveraging its full potential. The increasing integration of AI in healthcare settings has profound implications for the future of clinical practice.

## Language model use

The use of ChatGPT in this study was strictly limited to the response-generating protocol described in the methods section. ChatGPT was not used for data analysis, writing, or any other aspects of the production of this manuscript.

## Data Availability

The original contributions presented in the study are included in the article/Supplementary material, further inquiries can be directed to the corresponding author.
