# Supplementary material for: Large language model triaging of simulated nephrology patient inbox messages
Source: Front Artif Intell. 2024 Sep 9;7:1452469. doi: 10.3389/frai.2024.1452469 (PMC11417033; doi:10.3389/frai.2024.1452469)
Supplement: Supplementary file 1 [file Data_Sheet_1.pdf]

"Dr. Singh, I've noticed I'm not peeing as much as I used to, and I've got this uncomfortable feeling in my lower belly. I'm scared that my kidney disease is getting worse. Can you take a look?"

ChatGPT

ChatGPT

This message falls into the **urgent** category. The patient is experiencing a significant change in symptoms related to a pre-existing condition (kidney disease), which should be addressed within 48 to 72 hours.

"Dr. Patel, my leg has swollen to an alarming extent and the pain is excruciating to even the slightest touch. It has also taken on an unsettling discoloration. I'm deeply worried that something gravely serious is happening to me."

ChatGPT

ChatGPT

This message falls into the **emergency** category. The patient is experiencing severe pain, significant swelling, and discoloration, which could indicate a serious condition like deep vein thrombosis or an infection that requires immediate medical attention.

"Dr. Lee, I'm really worried about my blood pressure. It's been constantly high, around 160/100, even though I've been taking my pills every day like I'm supposed to. I'm afraid this could be really bad for my health in the long run. Can you help me?"

ChatGPT

ChatGPT

This message falls into the **urgent** category. The patient is experiencing consistently high blood pressure despite medication, which poses a significant health risk and should be addressed within 48 to 72 hours.

"Hey Dr. Sharma, I've been thinking about getting a medical alert bracelet. Can we discuss if this is necessary for my condition?"

ChatGPT

ChatGPT

This message falls into the **non-urgent** category. The patient is seeking advice about a medical alert bracelet, which can be discussed during a regularly scheduled appointment.

"Dr. Verma, I've been wondering if there are any clinical trials or new treatments that might be suitable for me. Can we explore these options?"

ChatGPT

ChatGPT

This message falls into the **non-urgent** category. The patient is inquiring about clinical trials or new treatments, which can be discussed during a regularly scheduled appointment.

"Dr. Sharma, I've been wondering if there are any specific dental considerations I should keep in mind as someone with kidney disease. Can we discuss this?"

ChatGPT

ChatGPT

This message falls into the **non-urgent** category. The patient is seeking information about dental considerations related to their kidney disease, which can be discussed during a regularly scheduled appointment.

"Hey Dr. Adams, I'm in a constant state of dizziness and on the verge of losing consciousness. My skin has turned pale and clammy. I'm utterly terrified and at a loss as to what's causing these symptoms."

ChatGPT

ChatGPT

This message falls into the **emergency** category. The patient is experiencing severe dizziness, near-fainting, and pale, clammy skin, which could indicate a serious and potentially life-threatening condition that requires immediate medical attention.

"Dr. Chakravarty, I've been thinking about the impact of my kidney disease on my bone health. Can we discuss ways to maintain strong bones?"

ChatGPT
ChatGPT

This message falls into the **non-urgent** category. The patient is seeking information about maintaining bone health in the context of kidney disease, which can be discussed during a regularly scheduled appointment.

"Dr. Bajaj, I've been having trouble with my balance and coordination. Is this a neurological problem caused by my kidney disease?"

ChatGPT
ChatGPT

This message falls into the **urgent** category. The patient is experiencing balance and coordination issues, which could indicate a neurological problem related to their kidney disease. This should be addressed within 48 to 72 hours.

"Hi Dr. Patel, I'm due for my annual kidney function tests. Can we schedule an appointment for next week?"

ChatGPT
ChatGPT

This message falls into the **non-urgent** category. The patient is requesting to schedule a routine appointment for annual kidney function tests, which can be arranged during regular scheduling.

"Hey Dr. Chopra, my hair and nails don't look healthy anymore. They're brittle and breaking easily. Could this be happening because my kidney disease is causing mineral imbalances in my body?"

ChatGPT
ChatGPT

This message falls into the **urgent** category. The patient is experiencing changes in hair and nail health, which could be related to mineral imbalances from kidney disease. This should be addressed within 48 to 72 hours.

"Dr. Chakravarty, I've been managing my diabetes well, but I want to ensure I'm doing everything I can to protect my kidneys. Can we review my treatment plan?"

ChatGPT

ChatGPT

This message falls into the **non-urgent** category. The patient is managing their diabetes and wants to review their treatment plan to protect their kidneys, which can be discussed during a regularly scheduled appointment.

"Hey Dr. Rao, I've been thinking about participating in a charity walk or fundraiser for kidney disease awareness. Can we discuss if this is appropriate for my current health status?"

ChatGPT

ChatGPT

This message falls into the **non-urgent** category. The patient is inquiring about participating in a charity event, which can be discussed during a regularly scheduled appointment.

"Dr. Dhillon, I've been feeling dizzy and lightheaded a lot. Could I be anemic because of my chronic kidney disease?"

ChatGPT

ChatGPT

This message falls into the **urgent** category. The patient is experiencing dizziness and lightheadedness, which could indicate anemia related to their chronic kidney disease. This should be addressed within 48 to 72 hours.

"Dr. Sharma, I'm having trouble controlling my bladder and I've had some accidents. Does this happen a lot to people with advanced kidney disease?"

ChatGPT

ChatGPT

This message falls into the **urgent** category. The patient is experiencing bladder control issues and accidents, which could be related to advanced kidney disease. This should be addressed within 48 to 72 hours.

"Dr. Rao, even though I'm being really careful with my diet, my electrolytes are still out of balance. I'm worried about what this could be doing to my body in the long run. Can you help me?"

ChatGPT

ChatGPT

This message falls into the **urgent** category. The patient is experiencing persistent electrolyte imbalances despite dietary efforts, which poses a significant health risk and should be addressed within 48 to 72 hours.

"Dr. Morris, I've suffered severe burns and as a consequence, my kidneys have ceased functioning. The doctor has stressed the urgent need for aggressive fluid therapy and immediate medical intervention. I'm in desperate need of your assurance that I will recover from this ordeal."

ChatGPT

ChatGPT

This message falls into the **emergency** category. The patient has suffered severe burns and subsequent kidney failure, requiring immediate medical intervention and aggressive fluid therapy.

"Hi Dr. Chen, I started taking this new medicine and now I've got a rash all over my body. I feel sick to my stomach and I can't stop throwing up. Could this be something called acute interstitial nephritis? That sounds scary."

ChatGPT
ChatGPT

This message falls into the **emergency** category. The patient is experiencing a widespread rash, nausea, and vomiting after starting a new medication, which could indicate a serious condition like acute interstitial nephritis that requires immediate medical attention.

”Hi Dr. Reddy, I've been thinking about starting a small garden to grow my own fresh produce. Can we discuss if this is a good idea for someone with kidney disease?"

ChatGPT
ChatGPT

This message falls into the **non-urgent** category. The patient is considering starting a garden and wants to discuss its suitability given their kidney disease, which can be addressed during a regularly scheduled appointment.

"Dr. Morgan, I'm in agonizing abdominal pain and I can't stop vomiting. Now, my kidneys are shutting down as well. I'm desperate to understand what's causing these critical symptoms."

ChatGPT
ChatGPT

This message falls into the **emergency** category. The patient is experiencing severe abdominal pain, persistent vomiting, and kidney failure, which require immediate medical attention to determine the cause and provide appropriate treatment.

"Dr. Kapoor, I've heard that stress can impact kidney health. Can we talk about stress management techniques at my next appointment?"

ChatGPT
ChatGPT

This message falls into the **non-urgent** category. The patient is interested in discussing stress management techniques related to kidney health, which can be addressed during a regularly scheduled appointment.

"Dr. Agarwal, I've been getting short of breath and my chest hurts. I'm scared that my kidney disease might be affecting my heart. What should I do?"

ChatGPT

ChatGPT

This message falls into the **emergency** category. The patient is experiencing shortness of breath and chest pain, which could indicate a serious cardiac issue potentially related to their kidney disease and requires immediate medical attention.

"Hey Dr. Sharma, I've been throwing up and there's blood in it. I'm also having trouble keeping any food down. I'm scared my kidney disease is causing problems with my digestive system. What should I do?"

ChatGPT

ChatGPT

This message falls into the **emergency** category. The patient is experiencing vomiting with blood and an inability to keep food down, which are serious symptoms that require immediate medical attention.

"Dr. Singh, I've been managing my hypertension well with the current medication. I'd like to discuss lifestyle changes that could further improve my blood pressure control."

ChatGPT

ChatGPT

This message falls into the **non-urgent** category. The patient is managing their hypertension well and wants to discuss lifestyle changes for further improvement, which can be addressed during a regularly scheduled appointment.

"Hey Dr. Yadav, my legs are swollen and red. I'm scared I might have a deep vein thrombosis. What should I do?"

ChatGPT

ChatGPT

This message falls into the **emergency** category. The patient is experiencing swollen and red legs, which could indicate a deep vein thrombosis, a serious condition that requires immediate medical attention.

"Hey Dr. Kapoor, I've been thinking about the impact of my kidney disease on my digestive health. Can we discuss ways to manage any related symptoms?"

ChatGPT

ChatGPT

This message falls into the **non-urgent** category. The patient wants to discuss the impact of kidney disease on their digestive health and ways to manage symptoms, which can be addressed during a regularly scheduled appointment.

"Hi Dr. Campbell, the pain I'm experiencing is beyond description and I'm completely unable to urinate. The doctor mentioned that my kidneys are shutting down. I'm confronted with the terrifying possibility that my life is in grave danger."

ChatGPT

ChatGPT

This message falls into the **emergency** category. The patient is experiencing severe pain and an inability to urinate, with the possibility of kidney failure, which requires immediate medical attention.

"Hey Dr. Young, I've been informed by the doctor that my blood test results are gravely concerning and urgent medical intervention is necessary. While I don't fully grasp the specifics of my condition, I understand the severity of the situation."

ChatGPT

ChatGPT

This message falls into the **emergency** category. The patient has received concerning blood test results and has been informed that urgent medical intervention is necessary, indicating a serious condition that requires immediate attention.

"Hey Dr. Verma, I've been considering taking a nutrition class to learn more about kidney-friendly cooking. Can we discuss if this would be beneficial for me?"

ChatGPT

ChatGPT

This message falls into the **non-urgent** category. The patient is considering taking a nutrition class and wants to discuss its benefits, which can be addressed during a regularly scheduled appointment.

"Dr. Reddy, I've been considering talking to a therapist to help cope with the emotional aspects of living with kidney disease. Can you recommend someone?"

ChatGPT

ChatGPT

This message falls into the **non-urgent** category. The patient is seeking a recommendation for a therapist to help cope with the emotional aspects of living with kidney disease, which can be discussed during a regularly scheduled appointment.

"Dr. Desai, I've noticed my skin looks different - the color has changed and it's really itchy all over. My doctor said this could mean my kidney disease is getting worse. Is that true?"

ChatGPT

ChatGPT

This message falls into the **urgent** category. The patient is experiencing changes in skin color and severe itching, which could indicate a worsening of their kidney disease. This should be addressed within 48 to 72 hours.

"Hi Dr. Bhasin, my joints are hurting and they're all swollen. Is this what they call renal osteodystrophy? Is it related to my kidney disease?"

ChatGPT

ChatGPT

This message falls into the **urgent** category. The patient is experiencing joint pain and swelling, which could be related to renal osteodystrophy associated with their kidney disease. This should be addressed within 48 to 72 hours.

"Hey Dr. Sanchez, I'm pregnant and I've suddenly been struck by debilitating headaches and severely impaired vision. My blood pressure has also spiked to an alarmingly high level. I'm consumed with worry about the potential impact on my unborn child."

ChatGPT

ChatGPT

This message falls into the **emergency** category. The patient is pregnant and experiencing severe headaches, impaired vision, and high blood pressure, which could indicate a serious condition like preeclampsia that requires immediate medical attention.

"Hey Dr. Mehta, I've been considering alternative therapies to complement my current treatment plan. Can we discuss the safety and effectiveness of these options?"

ChatGPT

ChatGPT

This message falls into the **non-urgent** category. The patient is interested in discussing alternative therapies to complement their current treatment plan, which can be addressed during a regularly scheduled appointment.

"Hi Dr. Gonzalez, I recently started a new antibiotic regimen and now my kidneys are failing rapidly. I'm overwhelmed with fear and uncertainty about what's happening to my body."

ChatGPT

ChatGPT

This message falls into the **emergency** category. The patient is experiencing rapid kidney failure after starting a new antibiotic regimen, which requires immediate medical attention.

"Dr. Sharma, I have a family history of polycystic kidney disease. Can we discuss screening options at my next appointment?"

ChatGPT

ChatGPT

This message falls into the **non-urgent** category. The patient wants to discuss screening options for polycystic kidney disease due to family history, which can be addressed during a regularly scheduled appointment.

"Dr. Arora, I'm having trouble breathing and my chest feels uncomfortable. Could this be happening because I have too much fluid in my body from my kidney failure?"

ChatGPT

ChatGPT

This message falls into the **emergency** category. The patient is experiencing difficulty breathing and chest discomfort, which could be due to fluid overload from kidney failure and requires immediate medical attention.

"Dr. Singh, I've been considering changing my living arrangements to better accommodate my health needs. Can we discuss the potential benefits and drawbacks of this?"

ChatGPT

ChatGPT

This message falls into the **non-urgent** category. The patient is considering changing their living arrangements to better accommodate their health needs, which can be discussed during a regularly scheduled appointment.

"Dr. Wallace, the pain I'm experiencing is excruciating. It's concentrated in my side and I'm vomiting uncontrollably. I've been running a high fever for several days and the antibiotics seem ineffective. I'm utterly exhausted and don't know how much more of this I can endure."

ChatGPT

ChatGPT

This message falls into the **emergency** category. The patient is experiencing severe side pain, uncontrollable vomiting, a high fever for several days, and ineffective antibiotics, indicating a serious condition that requires immediate medical attention.

"Hi Dr. Kapoor, I've been wondering if there are any specific vaccines I should get as someone with kidney disease. Can we review my vaccination status?"

ChatGPT

ChatGPT

This message falls into the **non-urgent** category. The patient is inquiring about vaccination recommendations for kidney disease, which can be addressed during a regularly scheduled appointment.

"Dr. Singh, I've been thinking about the long-term impact of my kidney disease. Can we discuss the potential progression and what I can expect in the future?"

ChatGPT

ChatGPT

This message falls into the **non-urgent** category. The patient is seeking to discuss the long-term impact and potential progression of their kidney disease, which can be addressed during a regularly scheduled appointment.

"Hey Dr. Saxena, I've noticed changes in how my pee looks and smells. It's darker and has a strong odor. Should I be worried?"

ChatGPT

ChatGPT

This message falls into the **urgent** category. The patient is experiencing noticeable changes in the appearance and odor of their urine, which could indicate a significant health issue that should be addressed within 48 to 72 hours.

"Hi Dr. Perez, I'm in unbearable agony and I'm shivering uncontrollably. My fever has spiked to an alarmingly high level. The severity of my condition has me fearing for my life. I'm begging for your immediate help."

ChatGPT

ChatGPT

This message falls into the **emergency** category. The patient is experiencing severe symptoms including unbearable agony, uncontrollable shivering, and a high fever, which require immediate medical attention due to the potential seriousness of their condition.

"Hi Dr. Reddy, I've noticed I'm having trouble remembering things and I can't focus like I used to. Could this be happening because of the toxins building up in my body from my kidney disease?"

ChatGPT

ChatGPT

This message falls into the **urgent** category. The patient is experiencing cognitive symptoms such as memory problems and difficulty focusing, which could be related to toxin buildup from kidney disease. This should be addressed within 48 to 72 hours to determine the cause and necessary interventions.

"Hey Dr. Harris, something's really wrong with me. My heart is pounding out of my chest and I can't think straight at all. The nurse mentioned my potassium levels are at a dangerous level. I'm terrified about what this could mean for me."

ChatGPT

ChatGPT

This message falls into the **emergency** category. The patient is experiencing severe symptoms including an elevated heart rate and confusion, combined with dangerously high potassium levels, which can be life-threatening and require immediate medical attention.

"Hey Dr. Clark, I'm in a constant state of breathlessness, no matter my efforts. My heart is palpitating at an alarmingly rapid pace, as if it might burst. The doctor mentioned my blood pH levels are severely imbalanced. I'm struggling to understand the gravity of this situation."

ChatGPT

ChatGPT

This message falls into the **emergency** category. The patient is experiencing severe symptoms including constant breathlessness, rapid heart palpitations, and significantly imbalanced blood pH levels, all of which suggest a potentially life-threatening condition that requires immediate medical intervention.

"Hi Dr. Bell, my lupus has taken a severe turn for the worse. I'm experiencing excruciating pain in my side and there's blood in my urine. The doctor has informed me that my kidneys are failing at an alarming rate. I'm absolutely terrified and in urgent need of your support."

ChatGPT

ChatGPT

This message falls into the **emergency** category. The patient is experiencing excruciating pain, blood in the urine, and rapid kidney failure related to their lupus condition. These symptoms are severe and potentially life-threatening, necessitating immediate medical attention.

"Hi Dr. Mehta, I've been wondering if there are any specific considerations I should keep in mind when choosing household cleaning products. Can we discuss this?"

ChatGPT

ChatGPT

This message falls into the **non-urgent** category. The patient is seeking advice on choosing household cleaning products, which is a general inquiry that can be addressed during a regularly scheduled appointment.

"Dr. Sharma, I've had kidney stones before and now my lower back is starting to hurt again. I really don't want to go through another kidney stone attack. Is there anything I can do to prevent it?"

ChatGPT

ChatGPT

This message falls into the **urgent** category. The patient has a history of kidney stones and is experiencing symptoms that may indicate a recurrence, such as lower back pain. It is important to address this concern within 48 to 72 hours to manage and potentially prevent another kidney stone attack.

"Dr. Lewis, I'm in a perpetual state of confusion and disorientation. My cognitive abilities are severely impaired and I'm struggling to articulate my thoughts. It's as if my mind is in complete disarray. I'm at a total loss as to what's happening to me."

ChatGPT

ChatGPT

This message falls into the **emergency** category. The patient is experiencing severe cognitive impairments, including confusion, disorientation, and difficulty articulating thoughts, which are serious symptoms that require immediate medical attention to determine the underlying cause and provide necessary treatment.

"Hi Dr. Menon, my testicles are swollen and tender. Could this be a sign of something wrong with my kidneys?"

ChatGPT

ChatGPT

This message falls into the **urgent** category. The patient is experiencing symptoms of swollen and tender testicles, which could be indicative of a serious underlying condition that needs to be evaluated and addressed within 48 to 72 hours to determine if it is related to kidney issues or another medical concern.

"Dr. Patel, I've been wondering if there are any specific precautions I should take when traveling to high altitudes. Can we discuss this?"

ChatGPT

ChatGPT

This message falls into the **non-urgent** category. The patient is inquiring about precautions for traveling to high altitudes, which is a general health inquiry that can be addressed during a regularly scheduled appointment.

"Hey Dr. Mehta, I've been reading about the benefits of a plant-based diet for kidney health. Can we go over my current diet and see if there are any improvements I can make?"

ChatGPT

ChatGPT

This message falls into the **non-urgent** category. The patient is interested in discussing potential dietary improvements, specifically regarding a plant-based diet for kidney health, which can be addressed during a regularly scheduled appointment.

"Hey Dr. Evans, I inadvertently ingested antifreeze and now my kidneys are rapidly deteriorating. I'm overcome with terror and I'm desperate for your immediate intervention and assistance."

ChatGPT

ChatGPT

This message falls into the **emergency** category. The patient has ingested antifreeze, which is a highly toxic substance that can cause rapid deterioration of kidney function and other severe health issues. Immediate medical intervention is crucial to address this life-threatening situation.

"Hey Dr. Carter, my skin and eyes have taken on a vivid yellow hue and I'm in constant, severe abdominal pain. My ability to urinate has diminished to almost nothing. The doctor's apparent concern for my well-being has me deeply troubled about my prognosis."

ChatGPT

ChatGPT

This message falls into the **emergency** category. The patient is experiencing symptoms indicative of severe liver issues or other critical conditions, such as jaundice (yellowing of the

skin and eyes), severe abdominal pain, and reduced urination. These symptoms require immediate medical attention to diagnose and treat the underlying causes.

"Hey Dr. Cook, I'm battling scleroderma and the pain has become unbearable. My blood pressure has reached dangerous heights and my kidney function is rapidly deteriorating. I'm consumed with fear about what the future holds for me."

ChatGPT

ChatGPT

This message falls into the **emergency** category. The patient is experiencing unbearable pain, dangerously high blood pressure, and rapid deterioration of kidney function related to scleroderma, a serious autoimmune disease. These symptoms suggest a critical and potentially life-threatening condition that requires immediate medical attention.

"Dr. Parker, I'm plagued by severe, debilitating muscle cramps and my heart rhythm is erratic, with frequent skipped beats. I've been informed that my magnesium levels have dropped to a critically low level. I'm struggling to understand the implications of this condition."

ChatGPT

ChatGPT

This message falls into the **emergency** category. The patient is experiencing severe muscle cramps, erratic heart rhythm, and critically low magnesium levels, which are urgent symptoms indicating potentially serious health issues, including cardiovascular risks. Immediate medical intervention is necessary to manage and treat these conditions.

"Dr. Verma, I've been thinking about the impact of my kidney disease on my mental health. Can we discuss strategies for maintaining a positive outlook and coping with any emotional challenges?"

ChatGPT

ChatGPT

This message falls into the **non-urgent** category. The patient is seeking to discuss the impact of kidney disease on their mental health and strategies for maintaining a positive outlook and

coping with emotional challenges, which can be addressed during a regularly scheduled appointment.

"Hi Dr. Sharma, I've been considering trying a new hobby to keep myself mentally engaged. Can we discuss if there are any activities I should avoid?"

ChatGPT

ChatGPT

This message falls into the **non-urgent** category. The patient is considering starting a new hobby and seeking advice on any activities to avoid, which can be discussed during a regularly scheduled appointment.

"Hi Dr. Verma, I've been experiencing occasional mild lower back pain. Could this be related to my kidney stones? I'd like to discuss prevention strategies."

ChatGPT

ChatGPT

This message falls into the **urgent** category. The patient is experiencing symptoms that may be related to kidney stones, such as occasional mild lower back pain. It is important to address this within 48 to 72 hours to discuss potential prevention strategies and evaluate the need for further medical intervention.

"Hi Dr. Chakravarty, I've been considering starting a blog or video channel to share my experiences with kidney disease. Can we discuss the potential benefits and drawbacks of this?"

ChatGPT

ChatGPT

This message falls into the **non-urgent** category. The patient is thinking about starting a blog or video channel to share their experiences with kidney disease, which is a topic that can be discussed during a regularly scheduled appointment to weigh the benefits and drawbacks.

"Hi Dr. Patel, I've been experiencing some mild hair loss. Could this be related to my kidney disease or medications? I'd like to discuss this at my next appointment."

ChatGPT
ChatGPT

This message falls into the **non-urgent** category. The patient is experiencing mild hair loss and is inquiring about potential causes related to kidney disease or medications. This issue can be addressed during a regularly scheduled appointment to discuss the possible links and any adjustments that may be needed.

"Hey Dr. Patel, lately I've been feeling more tired than usual and I've seen some blood in my pee. My regular doctor said something about my creatinine levels being a little high. What does that mean?"

ChatGPT
ChatGPT

This message falls into the **urgent** category. The patient is experiencing increased fatigue and blood in their urine, along with elevated creatinine levels, which could indicate a worsening of their kidney condition. This should be addressed within 48 to 72 hours.

"Dr. Varma, my phosphate levels are still high even though I'm taking phosphate binders. Could this be why I'm having bone problems?"

ChatGPT
ChatGPT

This message falls into the **urgent** category. The patient is experiencing high phosphate levels despite medication, which could be related to their bone problems and should be addressed within 48 to 72 hours.

"Hey Dr. Kim, I'm in agonizing pain when I attempt to urinate. At times, I'm completely unable to go, regardless of how strong the urge is. I sense there's a serious underlying issue, but I don't know what it could be."

ChatGPT
ChatGPT

This message falls into the **emergency** category. The patient is experiencing severe pain during urination and sometimes an inability to urinate, which could indicate a serious underlying issue that requires immediate medical attention.

"Dr. Chandra, my iron levels are low even though I'm taking supplements. I'm worried about how anemia could affect my overall health. What can I do?"

ChatGPT

ChatGPT

This message falls into the **urgent** category. The patient is experiencing low iron levels despite taking supplements, which could indicate persistent anemia that needs to be addressed within 48 to 72 hours.

"Dr. Gupta, I've been thinking about my long-term care options. Can we discuss advance directives and planning for the future?"

ChatGPT

ChatGPT

This message falls into the **non-urgent** category. The patient wants to discuss long-term care options and advance directives, which can be addressed during a regularly scheduled appointment.

"Dr. Joshi, I feel sick to my stomach all the time and I don't feel like eating anything. I'm worried that my kidney disease is messing with my digestive system. What can I do?"

ChatGPT

ChatGPT

This message falls into the **urgent** category. The patient is experiencing persistent nausea and loss of appetite, which could be related to their kidney disease and should be addressed within 48 to 72 hours.

"Hi Dr. Lee, I'm experiencing severe abdominal pain and there's a significant presence of blood in my urine. This is extremely frightening and I suspect there's a serious underlying problem. I'm greatly distressed by these symptoms."

ChatGPT

ChatGPT

This message falls into the **emergency** category. The patient is experiencing severe abdominal pain and significant blood in their urine, which could indicate a serious underlying problem that requires immediate medical attention.

"Hi Dr. Mishra, food doesn't taste or smell the same anymore. I don't enjoy eating like I used to. Is this a common problem for people with kidney disease?"

ChatGPT

ChatGPT

This message falls into the **urgent** category. The patient's experience of altered taste and smell and decreased enjoyment of eating could indicate an issue related to their kidney disease that should be addressed within 48 to 72 hours.

"Hey Dr. Choudhary, I keep getting these muscle cramps, especially at night. They're really painful. Is this because of my kidney disease?"

ChatGPT

ChatGPT

This message falls into the **urgent** category. The patient is experiencing frequent and painful muscle cramps, which could be related to their kidney disease and should be addressed within 48 to 72 hours.

"Dr. Mehta, I've been experiencing some sleep disturbances. Could this be related to my kidney function? I'd like to discuss ways to improve my sleep quality."

ChatGPT

ChatGPT

This message falls into the **non-urgent** category. The patient is experiencing sleep disturbances and wants to discuss potential links to their kidney function and ways to improve sleep quality, which can be addressed during a regularly scheduled appointment.

"Hi Dr. Rogers, ever since my cancer diagnosis, I've been experiencing severe pain in my side and my urine contains blood. My kidney function tests are also rapidly deteriorating. I'm overwhelmed with fear about what this means for my health."

ChatGPT

ChatGPT

This message falls into the **emergency** category. The patient is experiencing severe side pain, blood in their urine, and rapidly deteriorating kidney function following a cancer diagnosis, which requires immediate medical attention.

"Dr. Scott, ever since undergoing weight loss surgery, my kidneys have been functioning abnormally. I'm deeply distressed by this and I'm in desperate need of your help to understand what's wrong."

ChatGPT

ChatGPT

This message falls into the **urgent** category. The patient is experiencing abnormal kidney function following weight loss surgery, which could indicate complications that need to be addressed within 48 to 72 hours.

"Dr. Fletcher, I'm in a terrible state since my kidney transplant. I have an extremely high fever that's persisting and I'm in excruciating pain, especially around the transplant site. It's intensifying with each passing day. I'm deeply worried that something is seriously wrong."

ChatGPT

ChatGPT

This message falls into the **emergency** category. The patient is experiencing a persistent high fever and excruciating pain around the transplant site, which could indicate a serious

complication such as an infection or rejection of the transplanted kidney. Immediate medical attention is required.

"Dr. Hernandez, you know I have type 2 diabetes, right? Well, my blood sugar has been staying high even though I'm doing everything I'm supposed to do to control it. I'm really worried that this could be hurting my kidneys. What can I do?"

ChatGPT

ChatGPT

This message falls into the **urgent** category. The patient is experiencing consistently high blood sugar levels despite efforts to control it, which could negatively impact their kidneys and should be addressed within 48 to 72 hours.

"Dr. Chakravarty, I've been wondering about the genetic aspects of kidney disease. Can we discuss the chances of my children inheriting the condition?"

ChatGPT

ChatGPT

This message falls into the **non-urgent** category. The patient wants to discuss the genetic aspects of kidney disease and the chances of their children inheriting the condition, which can be addressed during a regularly scheduled appointment.

"Dr. Mehrotra, I'm having trouble peeing and I feel like my bladder doesn't empty all the way. Could I have a urological problem because of my kidney disease?"

ChatGPT

ChatGPT

This message falls into the **urgent** category. The patient is experiencing difficulty urinating and a feeling of incomplete bladder emptying, which could indicate a urological problem related to their kidney disease. This should be addressed within 48 to 72 hours.

"Dr. Nelson, I underwent a CT scan with contrast dye and subsequently, my kidneys have begun to shut down and I have not pee for the past 2 days. I'm struggling to comprehend the situation and I'm in desperate need of your expertise to help me understand what's going on."

ChatGPT

ChatGPT

This message falls into the **emergency** category. The patient is experiencing kidney shutdown and anuria (inability to urinate) following a CT scan with contrast dye, which requires immediate medical attention.

"Hey Dr. Gupta, I've been wondering if there are any specific considerations I should keep in mind when choosing personal care products. Can we discuss this?"

ChatGPT

ChatGPT

This message falls into the **non-urgent** category. The patient is inquiring about considerations for choosing personal care products, which can be discussed during a regularly scheduled appointment.

"Hi Dr. Moreno, I'm in a constant state of exhaustion and I'm plagued by relentless nausea. My skin has taken on a sickly yellow hue and my urine output has nearly ceased. I'm extremely frightened and I have no idea what's happening to me."

ChatGPT

ChatGPT

This message falls into the **emergency** category. The patient is experiencing extreme exhaustion, relentless nausea, jaundice (yellow skin), and nearly ceased urine output, which indicate a potentially life-threatening condition requiring immediate medical attention.

"Hey Dr. Turner, I was involved in a serious accident and the doctor has informed me that my kidneys have sustained significant damage as a result. I'm struggling to grasp the implications of this diagnosis and I'm in desperate need of your guidance to help me understand what this means for my health."

ChatGPT

ChatGPT

This message falls into the **emergency** category. The patient has sustained significant kidney damage from a serious accident and requires immediate medical attention and guidance to understand the implications of their diagnosis.

"Hi Dr. King, the pain I'm enduring is beyond description, it's absolutely unbearable. Moreover, my urine has taken on an extremely dark color, resembling cola. I'm utterly terrified by these developments."

ChatGPT

ChatGPT

This message falls into the **emergency** category. The patient is experiencing unbearable pain and dark-colored urine, which could indicate a serious underlying condition requiring immediate medical attention.

"Dr. Pandey, my magnesium levels are off even though I'm taking supplements. I'm scared this could affect my heart and nervous system. What should I do?"

ChatGPT

ChatGPT

This message falls into the **urgent** category. The patient's magnesium levels are abnormal despite taking supplements, which could affect the heart and nervous system. This should be addressed within 48 to 72 hours.

"Dr. Rao, I've been reading about the importance of exercise for kidney health. Can we discuss an appropriate exercise plan for me?"

ChatGPT

ChatGPT

This message falls into the **non-urgent** category. The patient wants to discuss an exercise plan for kidney health, which can be addressed during a regularly scheduled appointment.

"Hey Dr. Bailey, I recently received a new kidney, but now my urine output has nearly ceased and my blood test results are deeply concerning. The doctor suspects there may be a serious issue with the transplant. I'm filled with dread at the thought of losing my new kidney."

ChatGPT

ChatGPT

This message falls into the **emergency** category. The patient is experiencing a significant decrease in urine output and concerning blood test results following a kidney transplant, indicating a potential serious issue with the transplant that requires immediate medical attention.

"Hey Dr. Garg, even though I'm following a low-potassium diet, my potassium levels are still not right. I'm scared this could affect my heart rhythm. What can I do?"

ChatGPT

ChatGPT

This message falls into the **urgent** category. The patient is experiencing abnormal potassium levels despite following a low-potassium diet, which could affect their heart rhythm. This should be addressed within 48 to 72 hours.

"Dr. Murphy, my toes have turned blue and my kidneys are now failing. The doctor mentioned that cholesterol blockages in my blood vessels may be the culprit. I'm struggling to understand if this is indeed the underlying cause of my condition."

ChatGPT

ChatGPT

This message falls into the **emergency** category. The patient is experiencing blue toes and kidney failure, with a possible diagnosis of cholesterol blockages in blood vessels. This requires immediate medical attention to confirm the cause and provide appropriate treatment.

"Hi Dr. Baxter, I'm experiencing some frightening symptoms. There's a significant amount of blood in my urine and my body is severely swollen. My blood pressure is dangerously high. I'm extremely concerned and don't know how to handle this."

ChatGPT

ChatGPT

This message falls into the **emergency** category. The patient is experiencing significant blood in their urine, severe swelling, and dangerously high blood pressure, all of which are serious symptoms that require immediate medical attention.

"Dr. Edwards, I consumed wild mushrooms and as a result, I'm now hospitalized with acute kidney failure. The doctor suspects that I've been poisoned. I'm in desperate need of your reassurance about my chances for recovery."

ChatGPT

ChatGPT

This message falls into the **emergency** category. The patient is hospitalized with acute kidney failure due to suspected mushroom poisoning, which requires immediate medical attention and urgent reassurance and treatment.

"Dr. Roberts, I'm experiencing excruciating pain during urination and my fever is unrelenting. My urine is cloudy and emanating a foul odor. I'm convinced I've contracted a severe infection that requires urgent attention."

ChatGPT

ChatGPT

This message falls into the **emergency** category. The patient is experiencing severe pain during urination, a persistent fever, cloudy urine with a foul odor, indicating a likely severe infection that requires immediate medical attention.

"Dr. Duggal, I've noticed I'm coughing a bit and it's been somewhat difficult to breathe lately. Could this be related to pulmonary issues from my kidney condition?"

ChatGPT

ChatGPT

This message falls into the **urgent** category. The patient is experiencing coughing and difficulty breathing, which could be related to pulmonary issues from their kidney condition. This should be addressed within 48 to 72 hours.

"Hi Dr. Nguyen, I'm plagued by an excruciating pain in my chest that intensifies when I lie down, but slightly eases when I sit upright. This pain is unlike anything I've ever experienced before. I'm greatly concerned about the underlying cause."

ChatGPT

ChatGPT

This message falls into the **emergency** category. The patient is experiencing severe chest pain that worsens when lying down and eases when sitting upright, which could indicate a serious condition like pericarditis or another cardiac issue requiring immediate medical attention.

"Hi Dr. Singh, I've been wondering if there are any specific considerations I should keep in mind when choosing over-the-counter medications. Can we discuss this?"

ChatGPT

ChatGPT

This message falls into the **non-urgent** category. The patient is inquiring about considerations for choosing over-the-counter medications, which can be discussed during a regularly scheduled appointment.

"Dr. Allen, I'm constantly overwhelmed by exhaustion and my mental state is in a perpetual fog. I'm also experiencing severe abdominal pain and I've been told my calcium levels have reached a dangerously high level. This news is deeply alarming and I'm fearful of the potential consequences."

ChatGPT

ChatGPT

This message falls into the **emergency** category. The patient is experiencing severe exhaustion, mental fog, severe abdominal pain, and dangerously high calcium levels, which are serious symptoms that require immediate medical attention.

"Hey Dr. Gupta, I noticed a small amount of blood in my urine a few days ago. It hasn't happened since, but I'd like to get it checked out just in case."

ChatGPT

ChatGPT

This message falls into the **urgent** category. While the occurrence of blood in urine has stopped, it should still be checked out within 48 to 72 hours to rule out any potential underlying issues.

"Dr. Kaur, I keep getting UTIs. Could this be happening because of my diabetic nephropathy?"

ChatGPT

ChatGPT

This message falls into the **urgent** category. The patient is experiencing recurrent urinary tract infections (UTIs), which could be related to diabetic nephropathy and should be addressed within 48 to 72 hours.

"Dr. Phillips, I'm in a constant state of confusion and I'm experiencing frequent seizures. My blood pressure has also spiked to an alarmingly high level. I'm utterly terrified and I'm urgently seeking your help to understand what's happening to me."

ChatGPT

ChatGPT

This message falls into the **emergency** category. The patient is experiencing constant confusion, frequent seizures, and dangerously high blood pressure, which are serious symptoms that require immediate medical attention.

"Dr. Green, I'm afflicted by an excruciating pain in my side that refuses to subside. The intensity is escalating to an unbearable level. I'm convinced I need immediate hospitalization. I'm desperate for your guidance and assistance."

ChatGPT

ChatGPT

This message falls into the **emergency** category. The patient is experiencing escalating and unbearable side pain, indicating a potentially serious condition that requires immediate medical attention.

"Dr. Reddy, I'm interested in learning more about the latest advancements in treating chronic kidney disease. Can we discuss this at my next follow-up?"

ChatGPT

ChatGPT

This message falls into the **non-urgent** category. The patient wants to discuss the latest advancements in treating chronic kidney disease, which can be addressed during a regularly scheduled appointment.

"Hi Dr. Gupta, I've been considering joining a support group for people with kidney disease. Can we discuss the potential benefits of this?"

ChatGPT

ChatGPT

This message falls into the **non-urgent** category. The patient is interested in joining a support group for people with kidney disease and wants to discuss the potential benefits, which can be addressed during a regularly scheduled appointment.

"Hi Dr. Tandon, my hands and wrists are swollen and painful. Could I have amyloidosis because of my kidney failure?"

ChatGPT

ChatGPT

Urgent. This message describes symptoms like swelling and pain in the hands and wrists, possibly related to amyloidosis associated with kidney failure, suggesting it should be addressed within 48 to 72 hours to evaluate the cause and determine necessary interventions.

"Hey Dr. Reddy, I've been wondering if there are any new treatment options for my specific type of kidney disease. Can we review the latest research?"

ChatGPT

ChatGPT

Non-urgent. This message is about reviewing current research and potential treatment options for kidney disease, which can be discussed during a regularly scheduled appointment.

"Dr. Mitchell, the region surrounding my recently transplanted kidney is severely swollen and causing immense pain. I'm overwhelmed with worry that something has gone terribly wrong with the transplant. I urgently need you to assess the situation."

ChatGPT

ChatGPT

Emergency. This message indicates severe swelling and pain around a transplant site, suggesting potential complications that require immediate medical assessment and intervention to ensure the health of the transplanted kidney.

"Hey Dr. Nguyen, I haven't been feeling well lately. I've had a low fever and it really hurts when I pee - it's like a burning feeling. I'm thinking I might have a UTI. What should I do?"

ChatGPT

ChatGPT

Urgent. This message indicates symptoms of a urinary tract infection (UTI), such as low fever and painful urination. It should be addressed within 48 to 72 hours to confirm the diagnosis and start appropriate treatment.

"Hey Dr. Gill, my salivary glands are swollen and painful. Is this a rare complication of kidney disease?"

ChatGPT

ChatGPT

Urgent. This message describes swollen and painful salivary glands, which could be an unusual symptom needing medical evaluation to determine the cause and relationship to kidney disease. It should be addressed within 48 to 72 hours.

"Hi Dr. Gomez, even with my medication, I can't seem to get my blood pressure under control. And sometimes my heart feels like it's skipping beats. Could my kidney problems be causing this?"

ChatGPT

ChatGPT

Urgent. This message describes uncontrolled blood pressure and irregular heartbeats, potentially related to kidney issues. These symptoms should be addressed within 48 to 72 hours to evaluate the cardiovascular implications and adjust treatment as necessary.

"Dr. Nair, I've been experiencing some mild swelling in my ankles. Can we check my kidney function and discuss ways to manage this symptom?"

ChatGPT

ChatGPT

Urgent. This message indicates mild swelling in the ankles, which can be a sign of worsening kidney function or other underlying issues. It should be addressed within 48 to 72 hours to assess kidney function and discuss management strategies.

"Dr. Sinha, I feel weak all the time and my muscles are tired. Could I have a vitamin D deficiency because of my kidney disease?"

ChatGPT

ChatGPT

Urgent. This message reports chronic weakness and muscle fatigue, possibly due to vitamin D deficiency related to kidney disease. It should be addressed within 48 to 72 hours to assess vitamin levels and consider necessary dietary or supplement interventions.

"Dr. Rao, I've been considering making some changes to my work schedule to better manage my health. Can we discuss strategies for balancing work and my kidney disease management?"

ChatGPT

ChatGPT

Non-urgent. This message involves planning and discussing work-life balance to manage kidney disease, which can be addressed during a regularly scheduled appointment.

"Hi Dr. Bhatt, my weight is changing even though I'm not eating or exercising any differently. Could I be retaining fluid because of my kidney disease?"

ChatGPT

ChatGPT

Urgent. This message describes unexpected weight changes possibly due to fluid retention, which can be a symptom of worsening kidney disease. It should be addressed within 48 to 72 hours to evaluate the cause and adjust treatment if necessary.

"Hi Dr. Verma, I've been reading about the benefits of meditation for overall health. Can we discuss if this is something I should incorporate into my routine?"

ChatGPT

ChatGPT

Non-urgent. This message involves a general inquiry about incorporating meditation into a daily routine for health benefits, which can be discussed during a regularly scheduled appointment.

"Dr. Rivera, I've been coughing up a peculiar pink, frothy substance and my breathing is becoming increasingly labored. It feels as though my lungs are drowning in fluid. I'm utterly terrified by this development."

ChatGPT

ChatGPT

Emergency. This message describes symptoms such as coughing up a pink, frothy substance and labored breathing, suggesting acute pulmonary edema, a life-threatening condition that requires immediate medical attention.

"Dr. Patel, I've been considering taking a vacation, but I'm unsure how to manage my kidney disease while traveling. Can we discuss this at my next appointment?"

ChatGPT
ChatGPT
Non-urgent. This message pertains to discussing travel preparations and kidney disease management, which can be addressed during a regularly scheduled appointment.

"Dr. Nair, I've been taking my phosphate binders regularly, but I'm curious about other ways to manage my phosphate levels. Can we discuss this?"

ChatGPT
ChatGPT
Non-urgent. This message indicates a routine inquiry about managing phosphate levels and can be discussed during a regular appointment.

"Hi Dr. Goel, the color of my skin is changing and I'm getting these skin tags. Does this happen often to people with kidney disease?"

ChatGPT
ChatGPT
Urgent. This message suggests new symptoms that may require attention within 48 to 72 hours to assess any potential complications or underlying causes.

"Dr. Reed, I've just returned from a trip to Africa and now my kidneys are failing at an alarming rate. I'm overcome by weakness and I'm bruising at the slightest touch. The doctor suspects I may have contracted malaria. I'm terrified and in desperate need of your guidance on the severity of this diagnosis."

ChatGPT
ChatGPT
Emergency. This message indicates rapidly deteriorating kidney function, weakness, and easy bruising after returning from a trip, with a suspicion of malaria, suggesting immediate medical attention to confirm the diagnosis and begin appropriate treatment.

"Dr. Lopez, since beginning chemotherapy, I've been plagued by extreme fatigue and unrelenting vomiting. My urine output has diminished to a trickle. This seems to be a dire situation and I'm questioning if this is an expected part of the treatment process."

ChatGPT

ChatGPT

Emergency. This message describes severe symptoms including extreme fatigue, unrelenting vomiting, and significantly reduced urine output following chemotherapy, suggesting a potential severe reaction or complication that requires immediate medical attention.

"Dr. Martinez, I'm suffering from the most intense headaches imaginable and my vision is severely distorted. Everything appears hazy and out of focus. I've been informed by the doctor that my blood pressure has reached a dangerously high level. This sounds extremely alarming and I'm deeply concerned about the implications."

ChatGPT

ChatGPT

Emergency. This message describes symptoms of severe headaches, vision distortion, and dangerously high blood pressure, suggesting a potential hypertensive crisis that requires immediate medical attention to prevent serious complications.

"Hey Dr. Daniels, I'm struggling to breathe and it's absolutely terrifying. No matter what I do, I can't seem to get enough air. My legs have swollen to an alarming size, like they're about to burst. I've gained a tremendous amount of weight in such a short time. I'm really scared about what's happening to my body."

ChatGPT

ChatGPT

Emergency. This message indicates severe symptoms including difficulty breathing, extreme leg swelling, and rapid weight gain, which could suggest acute heart failure or other critical conditions requiring immediate medical attention.

"Hi Dr. Patel, I've been feeling a bit more fatigued lately. Can we check my anemia levels at my next appointment?"

ChatGPT

ChatGPT

Non-urgent. This message pertains to a routine check of anemia levels related to fatigue, which can be addressed during a regularly scheduled appointment.

"Dr. Kapoor, I've been considering switching to a different type of dialysis. Can we weigh the pros and cons of each option?"

ChatGPT

ChatGPT

Non-urgent. This message involves discussing potential changes to a dialysis regimen, which can be addressed during a regularly scheduled appointment to explore the benefits and drawbacks of different dialysis methods.

"Hi Dr. Gupta, I've been getting this numbness and tingling feeling in my hands and feet. Is this what they call uremic neuropathy? Is it because of my kidney disease?"

ChatGPT

ChatGPT

Urgent. This message indicates symptoms of numbness and tingling in the hands and feet, which could be related to uremic neuropathy associated with kidney disease. It should be addressed within 48 to 72 hours to confirm the diagnosis and discuss appropriate management strategies.

"Dr. Kapoor, I've been feeling really down and anxious recently. I can't help but wonder if it's because of my chronic kidney disease. What do you think?"

ChatGPT

ChatGPT

Urgent. This message indicates emotional distress potentially linked to chronic kidney disease, suggesting it should be addressed within 48 to 72 hours to evaluate and provide necessary mental health support.

"Hey Dr. Sethi, my periods have changed and I'm getting pelvic pain. Could my polycystic kidney disease be causing this?"

ChatGPT

ChatGPT

Urgent. This message describes changes in menstrual cycles and pelvic pain, which could potentially be related to complications from polycystic kidney disease. It should be addressed within 48 to 72 hours to evaluate the symptoms and determine any necessary interventions.

"Dr. Malhotra, I've noticed blood in my poop and I'm really worried. Could my kidney disease be causing internal bleeding somewhere?"

ChatGPT

ChatGPT

This message falls into the **urgent** category. The patient is experiencing blood in their stool, which could indicate internal bleeding potentially related to their kidney disease and should be addressed within 48 to 72 hours.

"Dr. Rao, I've been wondering if there are any specific precautions I should take when getting tattoos or piercings. Can we discuss this?"

ChatGPT

ChatGPT

This message falls into the **non-urgent** category. The patient wants to discuss precautions for getting tattoos or piercings, which can be addressed during a regularly scheduled appointment.

"Dr. Nair, I've been considering getting a pet for companionship and stress relief. Can we discuss if this is advisable for someone with my condition?"

ChatGPT
ChatGPT

This message falls into the **non-urgent** category. The patient is considering getting a pet for companionship and stress relief and wants to discuss its advisability, which can be addressed during a regularly scheduled appointment.

"Dr. Reynolds, the pain in my head is unbearable, it's blinding. My vision is severely blurred and the agony is indescribable. When I checked my blood pressure, it was alarmingly high, to a point where I thought the device was malfunctioning. I'm terrified I might be on the verge of a stroke or something equally severe."

ChatGPT
ChatGPT

This message falls into the **emergency** category. The patient is experiencing unbearable head pain, severely blurred vision, and alarmingly high blood pressure, which could indicate a serious condition such as a stroke and requires immediate medical attention.

"Dr. Gupta, I've been feeling so tired recently, more than usual. And my ankles and feet are swollen. I've also noticed my blood pressure is a bit higher than it normally is. Should I be worried?"

ChatGPT
ChatGPT

This message falls into the **urgent** category. The patient is experiencing unusual fatigue, swollen ankles and feet, and elevated blood pressure, which could indicate a worsening of their condition that should be addressed within 48 to 72 hours.

"Hey Dr. Verma, I'm having trouble seeing clearly and I keep seeing these floating spots. Could this be because of my high blood pressure and kidney problems?"

ChatGPT
ChatGPT

This message falls into the **urgent** category. The patient is experiencing vision problems, including floating spots, which could be related to high blood pressure and kidney problems. This should be addressed within 48 to 72 hours.

"Dr. Malik, this is kind of embarrassing, but I'm having trouble with sex. I don't have the same sex drive anymore. Could my chronic kidney disease be causing this?"

ChatGPT

ChatGPT

This message falls into the **urgent** category. The patient is experiencing a decrease in sex drive, which could be related to their chronic kidney disease and should be addressed within 48 to 72 hours.

"Hey Dr. Kim, I keep getting these headaches and my vision is blurry. My regular doctor said my blood pressure is too high and that I might need to change my medication. What do you think?"

ChatGPT

ChatGPT

This message falls into the **urgent** category. The patient is experiencing headaches, blurry vision, and high blood pressure, which may require a change in medication and should be addressed within 48 to 72 hours.

"Dr. Baker, I'm finding it increasingly difficult to breathe and my legs have swollen to an alarming degree. Additionally, my urine output has diminished to nearly nothing. I'm deeply concerned that these symptoms are interconnected and indicative of a serious underlying condition."

ChatGPT

ChatGPT

This message falls into the **emergency** category. The patient is experiencing difficulty breathing, severe leg swelling, and significantly reduced urine output, which could indicate a serious

underlying condition such as heart failure or kidney failure that requires immediate medical attention.

"Hey Dr. Gupta, I've been experiencing some dry and itchy skin. Is this related to my kidney disease? Can we talk about ways to manage this symptom?"

ChatGPT
ChatGPT

This message falls into the **urgent** category. The patient is experiencing dry and itchy skin, which could be related to their kidney disease. This should be addressed within 48 to 72 hours.

"Hi Dr. Hill, I'm experiencing vivid, inexplicable flashes of light and my vision is deteriorating at an alarming rate. Furthermore, my blood pressure has skyrocketed to a dangerously high level. I'm deeply troubled by these sudden changes in my health."

ChatGPT
ChatGPT

This message falls into the **emergency** category. The patient is experiencing vivid flashes of light, rapidly deteriorating vision, and dangerously high blood pressure, which could indicate a serious condition such as retinal detachment or hypertensive crisis requiring immediate medical attention.

"Dr. Stewart, I recently underwent a kidney transplant, but I'm now in severe pain and my urine output has diminished to nearly nothing. The doctor suspects there may be an obstruction affecting my new kidney. I'm hoping you can intervene and resolve this issue."

ChatGPT
ChatGPT

This message falls into the **emergency** category. The patient is experiencing severe pain and significantly reduced urine output after a kidney transplant, which could indicate an obstruction affecting the new kidney and requires immediate medical attention.

"Dr. Mehta, I'm having a hard time sleeping because my legs feel restless all the time. Someone told me this could be because of my kidney disease. Is that true? Can you help me get some relief?"

ChatGPT

ChatGPT

This message falls into the **urgent** category. The patient is experiencing restless legs, which could be related to their kidney disease, and should be addressed within 48 to 72 hours.

"Hey Dr. Nair, I've been wondering if there are any specific skincare routines I should follow as someone with kidney disease. Can we discuss this?"

ChatGPT

ChatGPT

This message falls into the **non-urgent** category. The patient is inquiring about specific skincare routines for someone with kidney disease, which can be discussed during a regularly scheduled appointment.

"Dr. Price, during my dialysis session, I was suddenly struck by a severe chest pain. It feels like there's an immense weight crushing down on me and I'm struggling to breathe. I'm desperately afraid I might be having a heart attack."

ChatGPT

ChatGPT

This message falls into the **emergency** category. The patient is experiencing severe chest pain and difficulty breathing during dialysis, which could indicate a heart attack or other serious condition requiring immediate medical attention.

"Hi Dr. Thompson, my muscles have become so weak, I can barely move a limb. I'm constantly plagued by odd sensations of tingling and numbness throughout my entire body. It's as if my body is gradually shutting down and I have no idea why."

ChatGPT

ChatGPT

This message falls into the **emergency** category. The patient is experiencing severe muscle weakness, tingling, and numbness throughout their body, which could indicate a serious underlying condition such as electrolyte imbalance, nerve damage, or a neurological disorder requiring immediate medical attention.

"Hey Dr. Wright, I'm experiencing severe muscular pain and an overwhelming sense of weakness. Additionally, my urine has darkened to a tea-like color. I'm incredibly fearful that my kidneys are in serious jeopardy."

ChatGPT

ChatGPT

This message falls into the **emergency** category. The patient is experiencing severe muscular pain, weakness, and darkened urine, which could indicate a serious condition such as rhabdomyolysis or kidney damage that requires immediate medical attention.

"Hey Dr. Singh, I've been considering trying yoga to help with my overall well-being. Is this safe for someone with kidney disease?"

ChatGPT

ChatGPT

This message falls into the **non-urgent** category. The patient is interested in trying yoga to improve their overall well-being and wants to discuss its safety in the context of their kidney disease, which can be addressed during a regularly scheduled appointment.

"Dr. Mehta, I've been thinking about the impact of my kidney disease on my sexual health. Can we discuss this in a sensitive and confidential manner?"

ChatGPT

ChatGPT

This message falls into the **non-urgent** category. The patient wants to discuss the impact of kidney disease on their sexual health in a sensitive and confidential manner, which can be addressed during a regularly scheduled appointment.

"Hi Dr. Patel, I've had this annoying pain in my lower back that just won't go away. It's been bothering me for a few days now. And when I pee, I've noticed some blood in it. I'm scared it might be a kidney stone. What do you think?"

ChatGPT

ChatGPT

This message falls into the **urgent** category. The patient is experiencing persistent lower back pain and blood in their urine, which could be indicative of a kidney stone or another serious condition. This should be addressed within 48 to 72 hours.

"Hi Dr. Chauhan, I've got this pain in my belly and I feel bloated. I'm worried that my polycystic kidney disease might be causing cysts to grow in other parts of my body too. Is that possible?"

ChatGPT

ChatGPT

This message falls into the **urgent** category. The patient is experiencing abdominal pain and bloating and is concerned that their polycystic kidney disease might be causing cysts to grow in other parts of their body. This should be addressed within 48 to 72 hours.

"Hi Dr. Collins, since commencing cancer treatment, I've been stricken with severe headaches and my vision is significantly blurred. Additionally, my kidney function is severely impaired. I'm deeply concerned that these issues are interconnected and I'm seeking your guidance to understand the potential link."

ChatGPT

ChatGPT

This message falls into the **emergency** category. The patient is experiencing severe headaches, significantly blurred vision, and impaired kidney function since starting cancer treatment, which could indicate a serious and potentially life-threatening condition requiring immediate medical attention.

"Dr. Tiwari, my calcium levels aren't right even though I'm taking supplements. I'm worried about what this could do to my bones and heart in the long run. Can you help me?"

ChatGPT
ChatGPT

This message falls into the **urgent** category. The patient is experiencing abnormal calcium levels despite taking supplements, which could affect their bones and heart and should be addressed within 48 to 72 hours.

"Hi Dr. Sharma, I've been adhering to a low-sodium diet, but I find it challenging to eat out. Can you provide some tips on making healthier choices when dining out?"

ChatGPT
ChatGPT

This message falls into the **non-urgent** category. The patient is seeking advice on making healthier choices when dining out while adhering to a low-sodium diet, which can be discussed during a regularly scheduled appointment.

"Hi Dr. Trivedi, I'm getting bruises and I'm bleeding under my skin. I'm worried that my kidney disease is affecting my blood's ability to clot properly. Is that possible?"

ChatGPT
ChatGPT

This message falls into the **urgent** category. The patient is experiencing bruising and bleeding under the skin, which could indicate a problem with blood clotting potentially related to their kidney disease. This should be addressed within 48 to 72 hours.
